# Lipocalin-type prostaglandin D synthase: a glymphopathy marker in idiopathic hydrocephalus

**DOI:** 10.3389/fnagi.2024.1364325

**Published:** 2024-04-04

**Authors:** Namiko Nishida, Nanae Nagata, Keigo Shimoji, Naoto Jingami, Kengo Uemura, Akihiko Ozaki, Makio Takahashi, Yoshihiro Urade, Sadayuki Matsumoto, Koichi Iwasaki, Ryosuke Okumura, Masatsune Ishikawa, Hiroki Toda

**Affiliations:** ^1^Department of Neurosurgery, Medical Research Institute Kitano Hospital, PIIF Tazuke-Kofukai, Osaka, Japan; ^2^Department of Animal Radiology, Graduate School of Agricultural and Life Sciences, The University of Tokyo, Tokyo, Japan; ^3^Department of Radiology, School of Medicine, Juntendo University, Tokyo, Japan; ^4^Department of Primary Care and Emergency Medicine, Graduate School of Medicine, Kyoto University, Kyoto, Japan; ^5^Department of Neurology, Graduate School of Medicine, Kyoto University, Kyoto, Japan; ^6^Department of Neurology, Osaka Red Cross Hospital, Osaka, Japan; ^7^Department of Neurodegenerative Disorders, Kansai Medical University, Osaka, Japan; ^8^Hirono Satellite, Isotope Science Center, The University of Tokyo, Fukushima, Japan; ^9^Department of Neurology, Medical Research Institute Kitano Hospital, PIIF Tazuke-Kofukai, Osaka, Japan; ^10^Department of Diagnostic Radiology, Medical Research Institute Kitano Hospital, PIIF Tazuke-Kofukai, Osaka, Japan

**Keywords:** diffusion tensor imaging, surface plasmon resonance, immunoprecipitation, western blotting, glymphopathy, tau, lipocalin-type prostaglandin D synthase, idiopathic normal pressure hydrocephalus

## Abstract

Idiopathic normal pressure hydrocephalus in elderly people is considered a form of glymphopathy caused by malfunction of the waste clearance pathway, called the glymphatic system. Tau is a representative waste material similar to amyloid-β. During neurodegeneration, lipocalin-type prostaglandin D synthase (L-PGDS), a major cerebrospinal fluid (CSF) protein, is reported to act as a chaperone that prevents the neurotoxic aggregation of amyloid-β. L-PGDS is also a CSF biomarker in idiopathic normal pressure hydrocephalus and significantly correlates with tau concentration, age, and age-related brain white matter changes detected by magnetic resonance imaging. To investigate this glymphopathy, we aimed to analyze white matter changes and contributing factors *in vivo* and their interactions *ex vivo*. Cerebrospinal tap tests were performed in 60 patients referred for symptomatic ventriculomegaly. Patients were evaluated using an idiopathic normal pressure hydrocephalus grading scale, mini-mental state examination, frontal assessment battery, and timed up-and-go test. The typical morphological features of high convexity tightness and ventriculomegaly were measured using the callosal angle and Evans index, and parenchymal white matter properties were evaluated with diffusion tensor imaging followed by tract-based spatial statistics. Levels of CSF biomarkers, including tau, amyloid-β, and L-PGDS, were determined by ELISA, and their interaction, and localization were determined using immunoprecipitation and immunohistochemical analyses. Tract-based spatial statistics for fractional anisotropy revealed clusters that positively correlated with mini-mental state examination, frontal assessment battery, and callosal angle, and clusters that negatively correlated with age, disease duration, idiopathic normal pressure hydrocephalus grading scale, Evans index, and L-PGDS. Other parameters also indicated clusters that correlated with symptoms, microstructural white matter changes, and L-PGDS. Tau co-precipitated with L-PGDS, and colocalization was confirmed in postmortem specimens of neurodegenerative disease obtained from the human Brain Bank. Our study supports the diagnostic value of L-PGDS as a surrogate marker for white matter integrity in idiopathic normal pressure hydrocephalus. These results increase our understanding of the molecular players in the glymphatic system. Moreover, this study indicates the potential utility of enhancing endogenous protective factors to maintain brain homeostasis.

## 1 Introduction

Idiopathic normal pressure hydrocephalus (iNPH) is treatable but can cause dementia, gait disturbance, and urinary incontinence in older patients with ventriculomegaly. The diagnosis of iNPH depends on successful cerebrospinal fluid (CSF) shunt surgery (Ishikawa et al., 2008; Mori et al., 2012; Nakajima et al., 2021). In addition to ventriculomegaly, brain imaging shows intraparenchymal white matter lesions, leukoaraiosis, and sometimes microbleeds, which may compromise brain function and the response to shunt therapy. Both leukoaraiosis and microbleeds are caused by impaired drainage of interstitial fluid and molecular clearance related to glymphatic (glia-lymphatic) system malfunction (Iliff et al., 2013; Weller et al., 2015; Simon and Iliff, 2016). Impaired glymphatic fluid transport resulting in the accumulation of intracellular waste products such as amyloid-β and tau aggregation is referred to as glymphopathy and may be involved in multiple pathological processes, including normal aging, Alzheimer’s disease, and iNPH (Benveniste and Nedergaard, 2022). We previously showed that the amount of lipocalin-type prostaglandin D synthase (L-PGDS) in CSF closely correlates with age-related brain white matter changes scale (Wahlund et al., 2001) detected via magnetic resonance imaging (MRI) of iNPH (Nishida et al., 2014).

The core symptoms of iNPH are non-specific. Thus, we considered diagnosing older adults with symptomatic ventriculomegaly using CSF tap tests (Ishikawa et al., 2010). Various biomarkers, including L-PGDS, were detected in CSF; however, the roles of these biomarkers in iNPH are unclear (Mase et al., 2003; Brettschneider et al., 2004). L-PGDS is a lipocalin family protein with multiple functions (Urade, 2021). In the central nervous system, L-PGDS is primarily synthesized in leptomeningeal cells and oligodendrocytes in rodents and humans and is secreted into the CSF as β-trace—a major human CSF protein (Chérasse et al., 2018; Urade, 2021). The L-PGDS expression is upregulated in oligodendrocytes in murine models of lysosomal disease (Mohri et al., 2006) and in the demyelinating focus of patients with multiple sclerosis (Kagitani-Shimono et al., 2006). L-PGDS promotes myelination by exocytosis from oligodendrocyte-lineage cells (Pan et al., 2023). In the context of neurodegeneration, L-PGDS acts as a chaperone, binding to amyloid β peptides to prevent fibril formation (Kanekiyo et al., 2007; Kannaian et al., 2019). We demonstrated that L-PGDS levels closely correlated with tau levels rather than amyloid β levels in CSF from patients with iNPH (Nishida et al., 2014).

The MRI diffusion tensor imaging (DTI) is frequently utilized to estimate white matter pathologies *in vivo* (Song et al., 2002; Alexander et al., 2007; Shimoji et al., 2013). Four parameters, including fractional anisotropy (FA), mean diffusivity (MD), axial diffusivity (AD), and radial diffusivity (RD), represent different aspects of white matter integrity and pathology, including axonal degeneration, and demyelination (Winklewski et al., 2018). The brain morphology of aged or hydrocephalic patients is not suitable for quantitative MRI analysis but methodological progress, including tract-based spatial statistics (TBSS), has been used to accomplish this task (Cader et al., 2007).

Thus, previous studies on normal pressure hydrocephalus reveal the close relationship between tau, L-PGDS, and MRI white matter damage scores. The recognized chaperone function of L-PGDS to amyloid-β—another key molecule in glymphopathy—indicates the relationship between tau and L-PGDS. We objectively assessed white matter damage by DTI analysis and identified relevant factors. Moreover, we confirmed whether tau directly interacts with L-PGDS. In this study, we analyzed the clinical, radiological, and CSF profiles of 60 consecutive patients who were referred to our institute with probable iNPH. We used TBSS parameters instead of age-related brain white matter changes to confirm the significant correlation of white matter integrity with L-PGDS levels in the CSF. Moreover, we demonstrate that L-PGDS binds to tau protein. Finally, we confirmed the colocalization of L-PGDS and tau protein in postmortem brain specimens. Based on our results, we hypothesize that L-PGDS works as a buffer for brain homeostasis by maintaining glymphatic drainage.

## 2 Materials and methods

### 2.1 Patients and clinical evaluations

Sixty patients diagnosed with probable or definite iNPH (mean age 76.0 ± 6.1 years; 37 males, 23 females) according to the Japanese guidelines (Ishikawa et al., 2008; Mori et al., 2012; Nakajima et al., 2021) were enrolled in this study. Before and 24 h after a CSF tap, gait, cognition, and incontinence were evaluated using the timed up-and-go test (TUG) (Podsiadlo and Richardson, 1991), the iNPH grading scale (iNPHGS) (Kubo et al., 2008), the mini-mental state examination (MMSE) (Folstein et al., 1975), and the frontal assessment battery (FAB) (Dubois et al., 2000).

#### 2.1.1 CSF analysis

Lumbar puncture was performed in the L3–L4 or L4–L5 interspace. CSF (10–30 mL) was collected and gently mixed to avoid gradient effects. CSF samples with cell count >5/mm^3^ were excluded. All CSF samples were centrifuged to remove cells and debris, aliquoted, and stored at −80°C until biochemical analysis. L-PGDS levels were measured with a standardized in-house ELISA, as previously described (Melegos et al., 1996). Tau and amyloid β levels were determined using commercially available ELISA kits (Invitrogen, Camarillo, CA, United States, and Immuno Biological Laboratories, Gunma, Japan, respectively). The control levels of L-PGDS, tau, and amyloid-β measured in our laboratories were previously reported (Nishida et al., 2014).

### 2.2 Purification of human recombinant Δ^1–22^ L-PGDS

Human recombinant Δ^1–22^ L-PGDS was prepared as previously described (Nagata et al., 2009). The coding region of human L-PGDS without the signal sequence at the amino-terminal (amino acid residues 1–22, defining the translation initiation codon Met as 1) was cloned into the pGEX-2 T vector (GE Healthcare, Amersham, Buckinghamshire, United Kingdom) to produce a fusion protein with GST. The plasmid was transformed into *E. coli* BL21 (DE3) (Urade et al., 1995). The recombinant GST-fusion protein was purified using glutathione-Sepharose 4B resin (GE Healthcare), followed by digestion with thrombin. The Δ^1–22^ L-PGDS was further purified by gel filtration chromatography using HiLoad Superdex 75 (GE Healthcare).

### 2.3 Surface plasmon resonance (SPR) analysis

The binding kinetics between human recombinant Tau-441 (WAKO) and recombinant human ∆^1–22^ L-PGDS were determined by SPR analysis using a Biacore 2000 system (GE Healthcare). Recombinant human L-PGDS was coupled to a CM5 sensor chip (GE Healthcare) by amine-coupling, according to the manufacturer’s protocol. The *K_d_* values were calculated from the sensorgrams using BIAevaluation 3.1 software (GE Healthcare).

### 2.4 Immunoprecipitation and western blotting

Human CSF was dissolved in Dulbecco’s phosphate-buffered saline containing protease inhibitors (Roche) and immunoprecipitated overnight at 4°C with 2 μg of anti-human L-PGDS antibody (Mab-10A5, Osaka Bioscience Institute). The sample was then incubated with 40 μL of protein G Sepharose 4 Fast Flow (GE Healthcare) for 2 h at 4°C. The resin was precipitated and washed three times with immunoprecipitation buffer. Proteins were eluted from the resin with 0.1 M glycine buffer (pH 2.5). Non-reduced sodium dodecyl surface-polyacrylamide gel electrophoresis followed by western blot analysis was performed to determine the protein expression of L-PGDS and tau. Protein samples were dissolved in 62.5 mM Tris-Cl (pH 6.8) containing 2% (w/v) SDS and 15% (v/v) glycerol, separated by 10–20% sodium dodecyl surface-polyacrylamide gel electrophoresis, and blotted onto polyvinylidine difluoride membranes (Immobilon P; Millipore, Bedford, MA, United States). After blocking in 5% non-fat milk for 1 h, the blots were incubated with primary antibodies (anti-human L-PGDS antibody, Osaka Bioscience Institute, or anti-tau antibody, Bioss, #bs-0419R) overnight at 4°C. The blots were then incubated with anti-rabbit IgG antibodies conjugated with horseradish peroxidase (GE Healthcare, #NA934V) at room temperature for 1 h. Immunoreactive signals were detected using the ECL Western Blotting Detection System (GE Healthcare).

### 2.5 Immunohistochemical analysis

Human brain sections from patients with Alzheimer’s disease were obtained from the Brain Bank (Platform of Supporting Cohort Study and Biospecimen Analysis, Platforms for Advanced Technologies, and Research Resources, Ministry of Education, Culture, Sports, Science, and Technology, Japan). Paraffin sections of formalin-fixed brains were examined. Deparaffinized and rehydrated sections (thickness, 10 μm) were pretreated with 0.3% (v/v) H_2_O_2_ in methanol for 30 min at room temperature and then incubated with 0.3% (w/v) pepsin (Sigma, St. Louis, MO, United States) in 0.01 N HCl for 5 min at room temperature for L-PGDS staining or autoclaved for 20 min for tau staining. The sections were then incubated with 5% (v/v) normal goat serum with 0.1% (v/v) Triton X-100 in PBS for 1 h at room temperature followed by incubation with rabbit anti-human L-PGDS antibody (Osaka Bioscience Institute) or mouse anti-tau antibody (Cell Signaling, Tokyo, Japan) overnight at 4°C. On the following day, the sections were incubated with anti-rabbit or anti-mouse IgG antibodies conjugated with biotin for 1 h at room temperature. After incubation with avidin-biotin complex (VECTASTAIN ABC Kit, Vector Laboratories, Burlingame, CA, United States) for 30 min at room temperature, the sections were incubated with 0.2 mg/mL 3,3′-diaminobenzidine tetrahydrochloride (DAB, Dojindo Laboratories, Kumamoto, Japan) and 0.03% hydrogen peroxidase. The images were captured using Aperio ScanScope XT (Leica, Tokyo, Japan). For double staining, after the primary antibodies, sections were sequentially incubated with Alexa Fluor 350-conjugated antibody against mouse IgG (Invitrogen) and Alexa Fluor 488-conjugated antibody against rabbit IgG (Invitrogen). To quench the autofluorescence associated with lipofuscin, immunostained sections were incubated in 1% Sudan Black B in 70% ethanol for 20 min. The images were captured using Scan scope (Aperio, Tokyo, Japan) and a BZ-X710 microscope (KEYENCE, Tokyo, Japan).

### 2.6 MRI analysis

A 3.0-T system (Achieva Quasar; Philips Medical Systems, Netherlands) was used for MRI. Three-dimensional T1-weighted fast field echo images (TR, 25 ms; TE, 2.2 ms; flip angle, 30°; slice thickness, 2.0 mm; intersection gap, 0.0 mm; field of view, 256 mm; matrix, 256 × 256; voxel size, 1.00 × 1.00 × 2.00 mm^3^), T2-weighted turbo spin echo images (TR, 6,000 ms; TE, 90 ms; flip angle, 90°; slice thickness, 2.0 mm; intersection gap, 0.0 mm; field of view, 256 mm; matrix, 256 × 256; voxel size, 1.00 × 1.00 × 2.00 mm^3^), and diffusion-weighted images (TR, 5,700 ms; TE, 65 ms; flip angle, 90°; slice thickness, 2.0 mm; intersection gap, 0.0 mm; field of view, 256 mm; matrix, 128 × 128; voxel size, 2.00 × 2.00 × 2.00 mm^3^) were obtained in axial sections covering the whole brain. Thirty-two diffusion directions were used for diffusion-weighted acquisition with and without diffusion encoding (*b* = 1,000 and 0 s/mm^2^). Ventriculomegaly and tight high convexity were assessed using structural MRI. The Evans index was calculated as the maximal width of the frontal horns/maximal width of the inner skull to approximate ventriculomegaly (Evans, 1942). The callosal angle was measured on the coronal image perpendicular to the anteroposterior commissure plane on the posterior commissure to approximate tight high convexity (Ishii et al., 2008).

### 2.7 Diffusion tensor imaging analysis

White matter integrity was evaluated by calculating FA, MD, AD, and RD with FSL version 6.0.3 (Smith et al., 2004). TBSS, part of FSL, was used for voxel-wise statistical analysis of FA and non-FA data (Cader et al., 2007). After eddy current correction, FA images were created by fitting a tensor model to the raw diffusion data using frequency doubling technology (from FSL), and non-brain tissue was deleted from the image using the brain extraction tool (Smith, 2002). The FA data from all patients were aligned in a common space using the non-linear registration tool FNIRT, which uses a b-spline representation of the registration warp field (Rueckert et al., 1999). Next, a mean FA image was created and thinned to a mean FA skeleton image representing the centers of all tracts common to the group. Each subject’s aligned FA data was projected onto this skeleton to obtain the skeletonized 4D data. Correlation analyses were performed by feeding the 4D data into voxel-wise cross-subject statistics. A linear regression model with a mask of the mean FA skeleton at the threshold value of 0.2 was used, and the number of permutations was set at 5,000. The threshold for significance was set at *p* < 0.05. The familywise error rate was corrected for multiple comparisons, using the threshold-free cluster enhancement option in the “randomize” permutation testing tool in FSL. We tested the correlation of each patient’s FA values with the following parameters; age, disease duration, opening pressure, Evans index (%), callosal angle, MMSE, FAB, TUG, iNPHGS, glucose, protein, tau, amyloid β, and L-PGDS using a general linear model matrix. Similar tests were performed for the MD, AD, and RD images. Finally, for the TBSS quality check, significant clusters were returned to native space with the tbss_deproject command to confirm their presence within the brain parenchyma. After visualization of significant clusters, the structures where the coordinates peak along with the associated probabilities were checked with the atlasquery/autaq command in FSL, using the Johns Hopkins University White-Matter Tractography Atlas (Wakana et al., 2007). To compare the structural distribution, voxel size was multiplied by the probabilities and shown in graphs for each parameter.

### 2.8 Standard protocol approvals and patient consent

This study was approved by the institutional review boards of Kitano Hospital (P151100704) and The University of Tokyo (approval number: 17–256). Informed consent was obtained from all participants. Human brain sections from patients with Alzheimer’s disease were available from the Brain Bank (Platform of Supporting Cohort Study and Biospecimen Analysis, Platforms for Advanced Technologies, and Research Resources, Ministry of Education, Culture, Sports, Science, and Technology, Japan) for qualified researchers under a collaborative and a tissue transfer agreement.

## 3 Results

### 3.1 Demographics

The clinical features of patients are summarized in Table 1. The definite iNPH group consisted of 30 shunt-responsive patients and the probable iNPH group consisted of 30 tap-test-responsive patients. Shunt placement depended on the decision of patients and caregivers. No significant differences in clinical profiles were detected between the two groups (Table 1). Thus, the two groups were combined for the MRI correlation analysis.

**Table 1:** Demographics of patients with probable or definite iNPH.

	Probable	Definite	*p-*value
Gender (m/f)	19/11	18/12	
Age	75.8 ± 7.0	76.2 ± 5.1	0.77
Disease duration	4.57 ± 3.2	4.40 ± 3.4	0.84
AchE inhibitor prescription	5	6	
Opening pressure (cmH_2_O)	13.2 ± 3.4	14.0 ± 4.1	0.45
Evans index (%)	33.1 ± 3.3	33.3 ± 2.6	0.79
Callosal angle (degree)	84.5 ± 18.9	84.0 ± 12.8	0.91
MMSE	20.6 ± 6.5	22.7 ± 5.6	0.20
FAB	11.2 ± 4.3	11.9 ± 3.6	0.45
TUG (sec)	21.4 ± 19.1	21.2 ± 12.8	0.96
TUG (step)	30.1 ± 21.8	28.9 ± 13.4	0.82
iNPHGS	7.30 ± 2.6	7.30 ± 2.4	0.99
Glucose (mg/dL)	78.8 ± 16.0	73.9 ± 12.5	0.19
Protein (mg/mL)	0.77 ± 0.16	0.76 ± 0.13	0.68
t-tau (pg/mL)	297.1 ± 263.0	251.8 ± 148.8	0.43
Aβ42 (pg/mL)	64.4 ± 31.2	56.2 ± 24.3	0.29
Aβ40 (pg/mL)	1,543 ± 473.1	1,360 ± 429.5	0.14
L-PGDS (μg/mL)	16.8 ± 4.4	16.3 ± 4.3	0.62

Data are actual numbers or means ± standard deviations. Aβ, amyloid-β; AchE, acetylcholinesterase; FAB, frontal assessment battery; iNPHGS, idiopathic normal pressure grading scale; L-PGDS, lipocalin-type prostaglandin D synthase; MMSE, mini-mental state examination; TUG, timed up-and-go test.

### 3.2 Interaction between tau and L-PGDS

**Figure 1:**
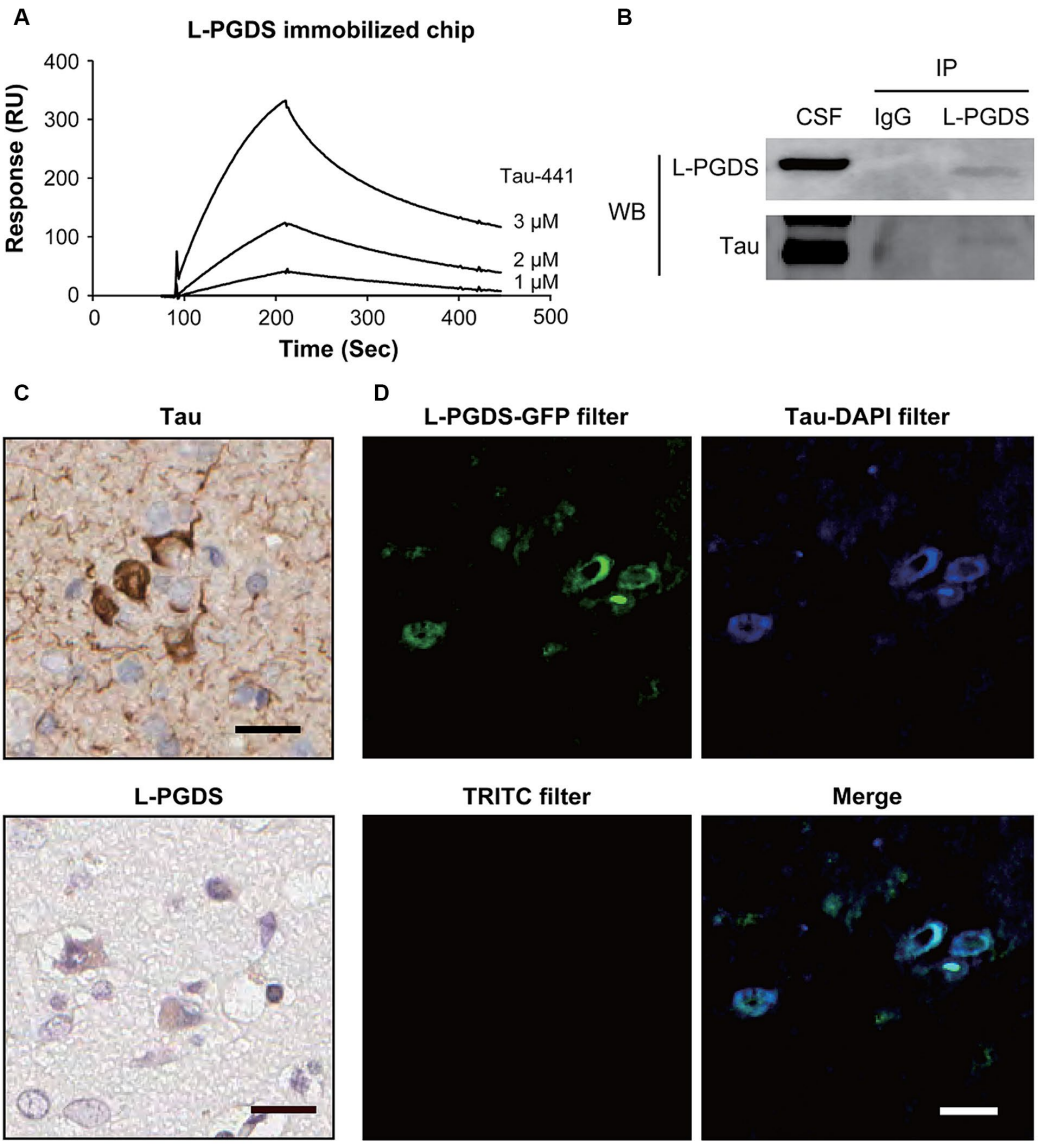
Interaction between Tau and L-PGDS. **(A)** Surface plasmon resonance (SPR) analysis of Tau-441 binding to lipocalin-type prostaglandin D synthase (L-PGDS). SPR sensorgrams of Tau-441 interaction with immobilized recombinant human ∆1–22 L-PGDS. Concentration of Tau-441 (from top to bottom): 3, 2, and 1 nM. **(B)** Co-immunoprecipitation between Tau and L-PGDS in human cerebral spinal fluid (CSF). Human CSF was subjected to immunoprecipitation with an anti-L-PGDS antibody, followed by Western blot analysis with anti-Tau and anti-L-PGDS antibodies. IP, immunoprecipitation; WB, Western blot. Results are representative of at least three independent experiments. **(C)** In the cortex of patients with Alzheimer’s disease, tau (upper) and L-PGDS (lower) were immunostained by anti-Tau and anti-human L-PGDS antibodies, respectively. **(D)** Double immunofluorescence images with Sudan Black B treatment. L-PGDS (green) was co-expressed with Tau-positive neurons (blue) in the cortex of patients with Alzheimer’s disease. The left lower panel shows an image with the TRITC filter. Scale bar 20 μm.

The SPR analysis was used to investigate the binding of L-PGDS to tau protein using a sensor chip with immobilized recombinant human ∆^1–22^ L-PGDS. In the human CNS, tau is expressed in six isoforms containing 352 to 441 residues. Tau-441 protein was used in the SPR analysis. As shown in Figure 1A, recombinant human Tau-441 protein bound to the immobilized L-PGDS in a dose-dependent manner and dissociated from L-PGDS when washed with Dulbecco’s phosphate-buffered saline. Tau-441 did not bind to immobilized bovine serum albumin (Supplementary Figure S1). Based on the association and dissociation curves, the *Kd* for Tau-441 was 0.91 mM. After identifying the weak interaction between recombinant L-PGDS and Tau-441 protein *in vitro*, we investigated this interaction *in vivo* via co-immunoprecipitation (Figure 1B and Supplementary Figure S2). Western blotting confirmed the protein expression of L-PGDS and tau in human CSF. Tau protein, observed around 70 kDa, was detected in immunoprecipitations with L-PGDS antibody but not with IgG isotype control antibody (Figure 1B and Supplementary Figure S2). These results suggest that the two proteins interact. In brain sections of patients with Alzheimer’s disease, tau signals were observed in neurons (Figure 1C). L-PGDS signals were observed in neurons, as described previously (Zhang et al., 2017). Double immunofluorescence staining revealed L-PGDS staining in tau-positive neurons in the cortex (Figure 1D). Staining of the double-immunolabeled sections with Sudan Black B blocked lipofuscin-associated autofluorescence of the sections (Figure 1D, lower left). These results suggest that L-PGDS may bind to tau protein.

### 3.3 Correlation of L-PGDS and white matter integrity

**Figure 2:**
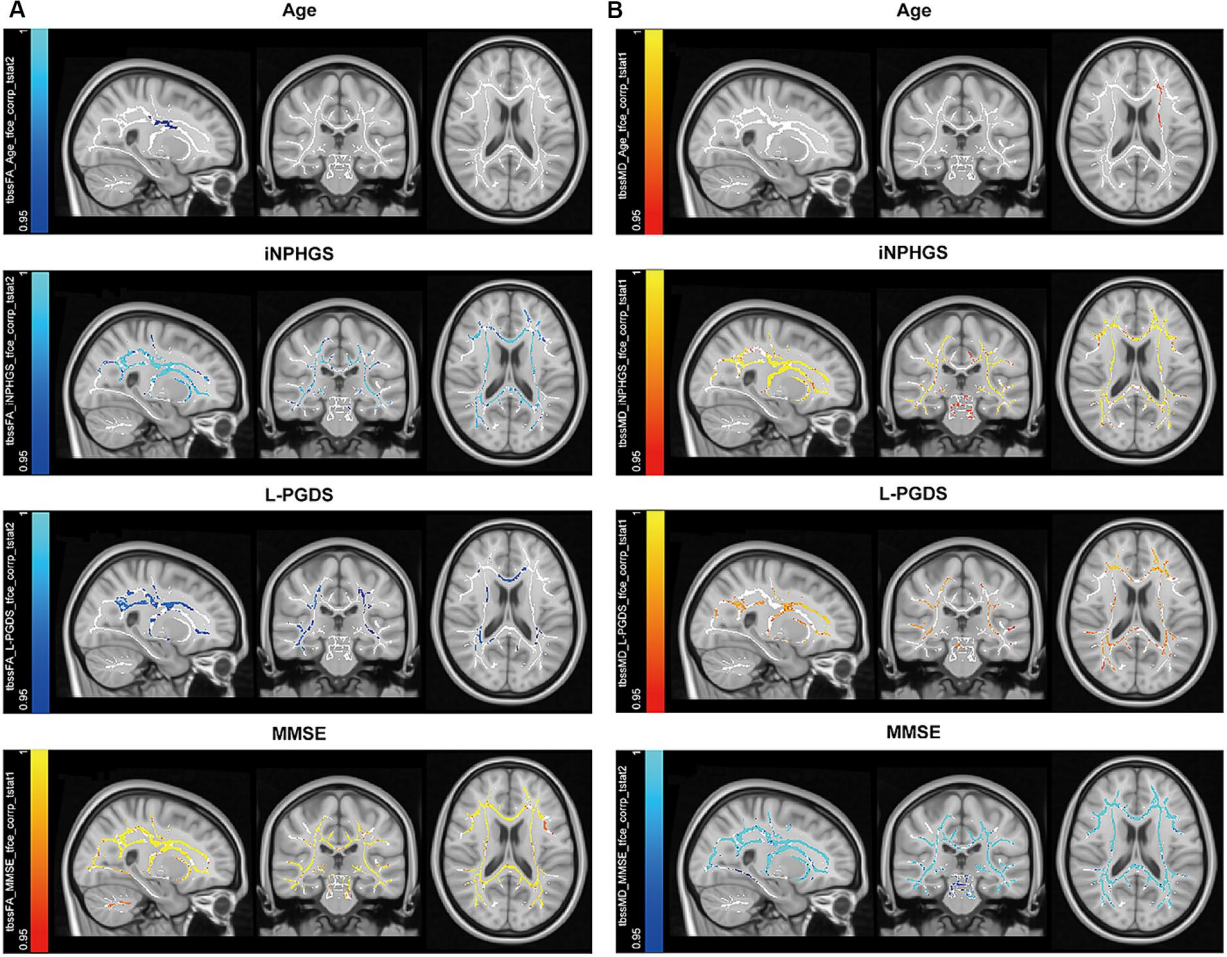
Correlated clusters with symptomatic, demographic, and cerebral spinal fluid biomarkers. **(A)** Negatively correlated clusters with fractional anisotropy are shown for age, idiopathic normal pressure hydrocephalus grading scale (iNPHGS), and lipocalin-type prostaglandin D synthase (L-PGDS) (cold color), while positively correlated clusters are shown for mini-mental state examination (MMSE) (warm color). **(B)** Positively correlated clusters with mean diffusivity are shown for age, iNPHGS, and L-PGDS (warm color), while negatively correlated clusters are shown for MMSE (cold color).

Correlations between the FA, MD, AD, RD distribution maps and the following parameters were determined: age, disease duration, opening pressure, Evans index (%), callosal angle, MMSE, FAB, TUG, iNPHGS, glucose, protein, tau, amyloid β, and L-PGDS using a general linear model of TBSS. FA clusters positively correlated with MMSE, FAB, and callosal angle, and negatively correlated with age, disease duration, iNPHGS, Evans index, and L-PGDS (Figure 2A). MD clusters positively correlated with age, iNPHGS, and L-PGDS, and negatively correlated with FAB and MMSE (Figure 2B). AD clusters positively correlated with iNPHGS and L-PGDS and negatively correlated clusters with age, Evans index, FAB, and MMSE. RD clusters positively correlated with age, disease duration, iNPHGS, and L-PGDS, and negatively correlated with FAB and MMSE. Age was relative non-corresponding to the brain region.

**Figure 3:**
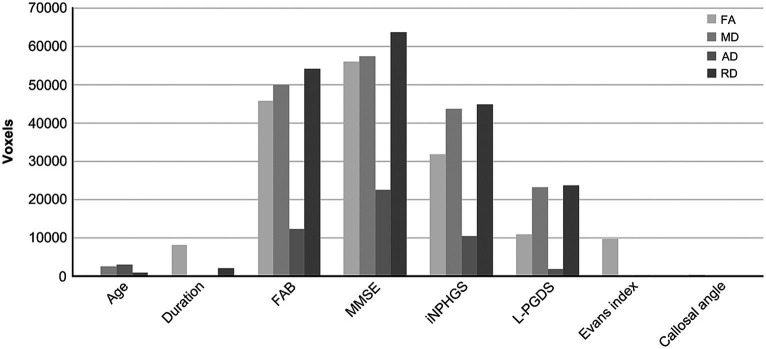
Total voxel counts correlated with each biomarker. Total voxel counts irrespective of correlation direction (i.e., positive or negative) are shown to compare the cluster size. Prominent voxel peaks are detected in frontal assessment battery (FAB), mini-mental state examination (MMSE), idiopathic normal pressure hydrocephalus grading scale (iNPHGS), and lipocalin-type prostaglandin D synthase (L-PGDS). AD, axial diffusivity; FA, fractional anisotropy; MD, mean diffusivity; RD, radial diffusivity.

**Figure 4:**
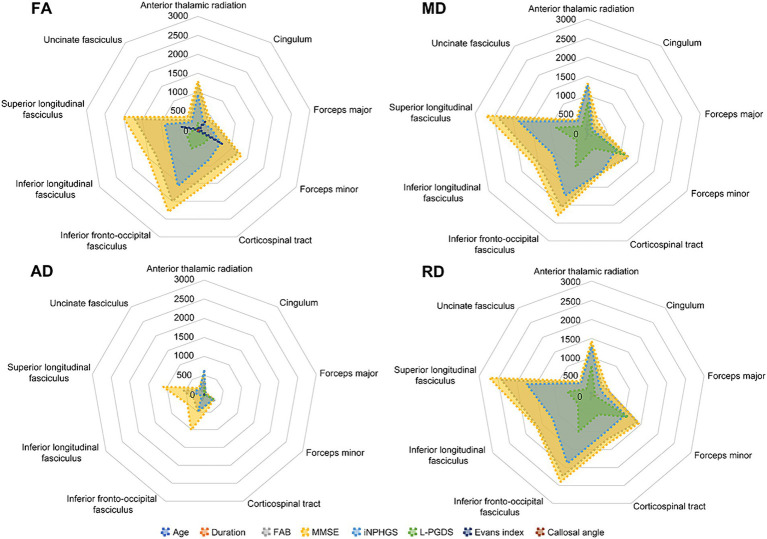
Cluster localizations probabilities in 20 structures of Johns Hopkins University White-Matter Tractography Atlas. Cluster localizations were shown in radar charts estimated by the products of voxel size multiplied by the probabilities of affiliation for 20 structures identified in the Johns Hopkins University White-Matter Tractography Atlas. Most of affected white matter tracts are association fibers including inferior fronto-occipital fasciculus and superior longitudinal fasciculus, and commissural fibers such as forceps major and minor. AD, axial diffusivity; FA, fractional anisotropy; FAB, frontal assessment battery; iNPHGS, idiopathic normal pressure hydrocephalus grading scale; L-PGDS, lipocalin-type prostaglandin D synthase; MD, mean diffusivity; MMSE, mini-mental state examination; RD, radial diffusivity.

To compare the impact of cluster size, total voxel counts irrespective of correlation direction (positive or negative) are shown in Figure 3. Prominent voxel peaks were identified for FAB, MMSE, iNPHGS, and L-PGDS. Cluster localizations are shown in radar charts estimated by the products of voxel size and the probabilities of affiliation for 20 structures identified in the Johns Hopkins University White-Matter Tractography Atlas (Figure 4). Most of the affected white matter tracts were association fibers, including the inferior fronto-occipital fasciculus and the superior longitudinal fasciculus, and commissural fibers, including the forceps major, and minor.

For comparison, previous analyses of the relationships between biomarkers of iNPH and the four DTI parameters are summarized in Table 2 (Hattingen et al., 2010; Koyama et al., 2013; Kamiya et al., 2016). The review data on Alzheimer’s disease was added for CSF biomarkers (Alm and Bakker, 2019). Existing reports on iNPH primarily mention the relationship between symptomatic severity and white matter integrity. The trends observed for each clinical parameter shared common features with our study. For CSF neurodegenerative biomarkers, Alzheimer’s disease cohorts were extensively studied, and tau and amyloid β correlate with DTI parameters in opposite directions. The correlation between tau or amyloid β and DTI parameters have similarly been investigated in cases of iNPH, but no significant results have been observed in previous reports or in our cohort. However, L-PGDS had significantly correlated clusters with DTI parameters in our cohort, and the positive and negative directions of the correlation were similar to that of tau in the Alzheimer’s disease cohorts.

**Table 2:** Relationship between biomarkers and diffusion tensor imaging (DTI) parameters in iNPH and Alzheimer’s disease.

Category		FA	MD	AD	RD	*N*	Year	Author
	Normal pressure hydrocephalus							
Symptoms	Walking steps	Neg.	Pos.			11	2010	Hattingen et al.
	MMSE	Pos.	Neg.					
	Gait of iNPHGS	Neg.				24	2013	Koyama et al.
	MMSE	Pos.				29	2016	Kamiya et al.
	FAB	Pos.						
	TMT-A^−1^	Pos.	Neg.		Neg.			
	iNPHGS	Neg.	Pos.	Pos.	Pos.	60	2023	Present study
	MMSE	Pos.	Neg.	Neg.	Neg.			
	FAB	Pos.	Neg.	Neg.	Neg.			
Morphology	Evans index	Neg.		Neg.				
	Callosal angle	Pos.						
Demographics	Age	Neg.	Pos.	Neg.	Pos.			
	Duration	Neg.			Pos.			
CSF	L-PGDS	Neg.	Pos.	Pos.	Pos.			
	Alzheimer’s disease							
	Amyloid β	Pos.	Neg.	Neg.	Neg.	192	2019	Alm and Bakker
	Tau	Neg.	Pos.	Pos.	Pos.			
Structural changes suggested by the direction of correlation								
Axonal degeneration		Neg.	Pos.	Neg.	Pos.			
Axonal packing		Pos.	Neg.	Pos.				
Demyelination		Neg.	Pos.		Pos.			

AD, axial diffusivity; CSF, cerebrospinal fluid; FA, fractional anisotropy; FAB, frontal assessment battery; iNPHGS, idiopathic normal pressure hydrocephalus grading scale; L-PGDS, lipocalin-type prostaglandin D synthase; MD, mean diffusivity; MMSE, mini-mental state examination; Neg., negative correlation; Pos., positive correlation; RD, radial diffusivity; TMT-A, trail-making test-A.

## 4 Discussion

We investigated the relationship between clinico-radiological factors and CSF profiles of iNPH patients using DTI analysis, with particular attention to parenchymal white matter integrity *in vivo* and the interaction of tau and L-PGDS *ex vivo*. We focused on L-PGDS due to its chaperone activity (including amyloid β), which may play a role in glymphopathy in iNPH. Our results highlight two main findings. First, we confirmed the significant correlation of several DTI parameters with clinico-demographical parameters and CSF L-PGDS levels. The main CSF trends were as follows: increased L-PGDS negatively correlated with overall white matter integrity (FA) and positively correlated with markers of demyelination (MD and RD). Most of the affected white matter tracts are long traveling association and commissural fibers. Second, we demonstrated that L-PGDS binds to tau protein, suggesting that L-PGDS functions as a tau chaperone in a similar fashion to how it functions with amyloid β (Kanekiyo et al., 2007; Kannaian et al., 2019). We also confirmed the colocalization of L-PGDS with tau in brain sections from patients with Alzheimer’s disease, a representative glymphopathy.

Many differential diagnoses exist for iNPH due to the non-specific symptoms, including gait, urination, and cognition frequently in aging. Tight high convexity and disproportionately enlarged subarachnoid-space hydrocephalus (DESH) are important features of ventricular enlargement distinguishing cerebral atrophy from impaired CSF circulation. The mechanism by which impaired CSF circulation leads to DESH morphology is still unknown, although a check valve phenomenon around the choroidal fissure has been proposed (Yamada et al., 2016). CSF shunting will reduce this tightness in high convexity. Of note, DESH indicates the contours of changes in the brain, but the acceptable extent of brain parenchymal white matter impairment is unclear. A mixture of axonal degeneration and demyelination are among the changes in the brain parenchyma of normal pressure hydrocephalus (Tullberg et al., 2002). FA clusters correlating with L-PGDS may reflect demyelination indicated by MD/RD rather than axonal degeneration indicated by AD. L-PGDS is secreted by oligodendrocytes and involved in demyelinating diseases and myelination (Kagitani-Shimono et al., 2006; Chérasse et al., 2018; Pan et al., 2023). Notably, L-PGDS concentration in CSF correlates with changes suggesting demyelination in TBSS. Indeed, shunt surgical intervention and subsequent recovery of brain parenchymal structure and function has several unexplored aspects. The reversibility of brain dysfunction by shunting should reflect the degree of brain parenchymal tissue damage, but cases with extensive white matter damage and microbleeds should be considered carefully, even if the brain contour is DESH. Increased microbleeds correlate with ventricular enlargement in Alzheimer’s disease (Kuroda et al., 2020). As for DESH brain, parenchymal atrophy seems to advance over the years, gradually losing its characteristic sulcal narrowness while symptoms progress (Toma et al., 2011; Jaraj et al., 2017; Isaacs and Hamilton, 2021).

Glymphopathy caused by malfunction of waste clearance in the central nervous system occurs in iNPH (Ringstad et al., 2017; Benveniste et al., 2019; Benveniste and Nedergaard, 2022). A well-known disease model of glymphopathy is Alzheimer’s disease, one of the major comorbidities for iNPH. Distinguishing between the two diseases is difficult, especially in the advanced stages. Although correlations with DTI parameters in both diseases have been reported, white matter fibers that correlate with CSF amyloid β and tau have been detected for Alzheimer’s disease but not for iNPH (Hattingen et al., 2010; Koyama et al., 2012; Kamiya et al., 2016; Alm and Bakker, 2019). These results are similar to the results from our study; we did not detect any white matter tract correlations with CSF biomarkers related to neurodegeneration, such as amyloid β and tau, in patients with iNPH. However, we did find fibers that correlated with L-PGDS, suggesting that this molecule plays a significant role in the disease.

In neurodegeneration, amyloid β fibril formation is prevented by the chaperone activity of L-PGDS (Kanekiyo et al., 2007; Kannaian et al., 2019). However, another important biomarker of neurodegenerative disease, tau, has not been studied. Tau is a hydrophilic protein but also forms neurotoxic aggregates in Alzheimer’s disease and in several tauopathies (Mandelkow and Mandelkow, 2012). We previously showed that tau levels were closely linked with L-PGDS levels in the CSF of patients with iNPH (Nishida et al., 2014). Tau carries many charges and can interact with many partners, yet its interaction with L-PGDS has not been clarified. Accordingly, we determined the interaction between the tau and L-PGDS *ex vivo* and found that the interactions were similar to those observed with amyloid β. Because L-PGDS can adsorb hydrophobic small molecules owing to the lipocalin capacity, tau is also adsorbed intracellularly. During a study involving the staining of monkey kidneys, fluorescent signals from labeled L-PGDS and lysosomes were merged (Nagata et al., 2009), suggesting that L-PGDS adsorbs tau, which is then degraded in lysosomes. Potentially, L-PGDS removes tau from the cell by adsorbing this for lysosomal degradation. Thus, L-PGDS may play a role in waste clearance pathways, such as the glymphatic system.

The CSF biomarker tau may be involved in the progression of iNPH (Kudo et al., 2000). Tau, a microtubule-associated protein, is primarily located in the axons of neuronal cells that promotes and stabilizes microtubule assembly (Weingarten et al., 1975; Mandelkow and Mandelkow, 2012). Increased tau levels in CSF correlate with the severity of neuronal damage and loss (Blennow et al., 1995). Tau increases in the CSF with age and the severity of clinical symptoms in iNPH (Kudo et al., 2000; Nishida et al., 2014). In contrast, tau levels tend to be low in patients with good cognitive recovery following shunt surgery (Kudo et al., 2000; Miyajima et al., 2013).

Decreased L-PGDS may be due to the arachnoidopathy (loss of arachnoid cells producing L-PGDS) secondary to NPH after subarachnoid hemorrhage (Mase et al., 2003; Brettschneider et al., 2004). Decreased L-PGDS correlated with a narrow callosal angle, which is a feature of uneven CSF distribution in DESH-type iNPH (Nishida et al., 2014). Paradoxically, patients with low L-PGDS concomitant with low tau levels exhibited high cognitive function despite severe arachnoidopathy. The role of arachnoidopathy in the development of DESH-type iNPH is unclear. One hypothesis is that L-PGDS bound to tau remains within the brain parenchyma due to stagnated glymphatic flow while the canopy is narrow in the prodromal to early phases of DESH in iNPH. After disease progression and brain atrophy progression, L-PGDS is released into the CSF and can be detected by lumber puncture.

Another discrepancy is the difference in location, with immunohistochemical studies showing colocalization of L-PGDS and tau usually in the gray matter, whereas MRI evaluation captures white matter damage partially due to the methodology of the MRI studies. However, this can be interpreted as immunohistochemical studies capturing the adsorption of pathological tau by L-PGDS in gray matter cells. Considerably, both substances increase in the CSF as the cellular degeneration progresses, causing progressive white matter damage on MRI and producing more severe symptoms.

Our study has several limitations. We did the histopathological study on brain specimens with Alzheimer’s disease instead of iNPH, due to difficulties in obtaining postmortem brain tissue from patients with iNPH. We demonstrated the interaction between L-PGDS and tau by SPR analysis, but the regulation of tau by L-PGDS is hard to demonstrate *in vitro*. Moreover, we included both definite iNPH and probable iNPH patients with various stages of disease for our *in vivo* analysis.

In conclusion, our findings support a role for L-PGDS in iNPH pathogenesis or progression, based on the concept of glymphopathy. The detailed role of L-PGDS is still unclear, but one possibility is that the chaperone activity of L-PGDS inhibits tau aggregation. Although iNPH is still an obscure heterogeneous disease concept, we hope our findings clarify the cause of this disease and aging brain homeostasis in general.

## Data availability statement

The original contributions presented in the study are included in the article/Supplementary material, further inquiries can be directed to the corresponding author/s.

## Ethics statement

The studies involving humans were approved by Institutional review boards of Kitano Hospital (P151100704) and the University of Tokyo (approval number: 17-256). The studies were conducted in accordance with the local legislation and institutional requirements. The participants provided their written informed consent to participate in this study.

## Author contributions

NNi: Writing–original draft, Writing – review & editing. NNa: Writing–original draft, Writing – review & editing. KS: Methodology, Writing – review & editing, Writing–original draft. NJ: Data curation, Writing – review & editing, Writing – original draft. KU: Methodology, Writing – review & editing, Writing – original draft. AO: Methodology, Writing – review & editing, Writing – original draft. MT: Methodology, Writing – review & editing, Writing – original draft. YU: Conceptualization, Writing – review & editing, Writing – original draft. SM: Validation, Writing – review & editing, Writing – original draft. KI: Validation, Writing – review & editing, Writing – original draft. RO: Data curation, Writing – review & editing, Writing – original draft. MI: Supervision, Validation, Writing – review & editing, Writing – original draft. HT: Supervision, Validation, Writing – review & editing, Writing – original draft.
