# Supplementary figures and images for: Lipocalin-type prostaglandin D synthase: a glymphopathy marker in idiopathic hydrocephalus
Source: Front Aging Neurosci. 2024 Apr 4;16:1364325. doi: 10.3389/fnagi.2024.1364325 (PMC11024442; doi:10.3389/fnagi.2024.1364325)

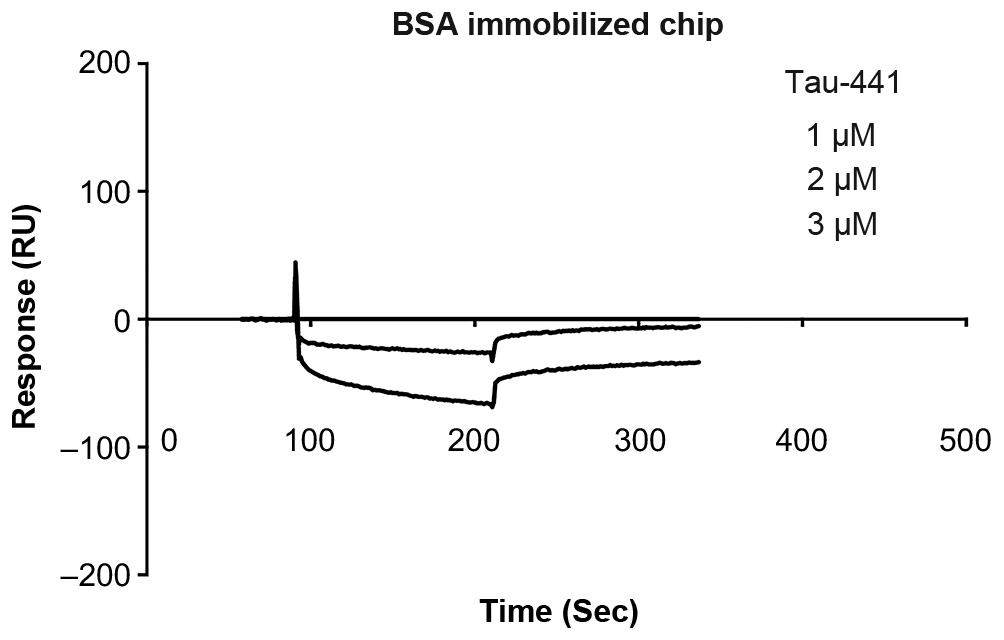

Supplement: Supplementary file 1 [file Image_1.TIF]

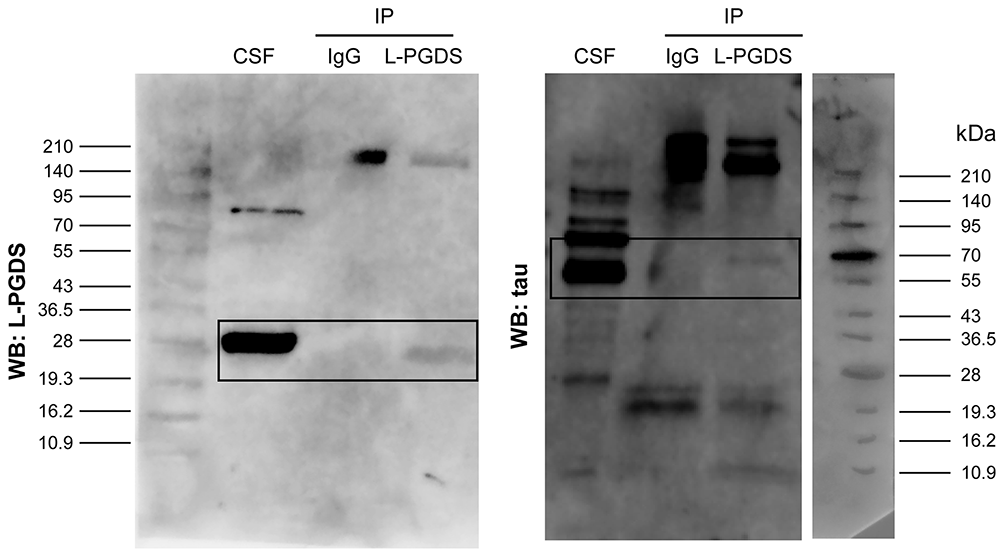

Supplement: Supplementary file 2 [file Image_2.TIF]
